# Impact of PSA density of transition zone as a potential parameter in reducing the number of unnecessary prostate biopsies in patients with PSA levels between 2.6 and 10.0 ng/mL

**DOI:** 10.1590/S1677-5538.IBJU.2017.0506

**Published:** 2018

**Authors:** Hugo A. Socrates Castro, Wagner Iared, José Eduardo Mourão Santos, Raphael Sandes Solha, David Carlos Shigueoka, Sergio Aron Ajzen

**Affiliations:** 1 Departamento de Diagnóstico por Imagem, Universidade Federal de São Paulo, Unifesp, São Paulo, SP, Brasil; 2 Departamento de Radiologia, Universidade Federal de São Paulo, Unifesp, São Paulo, SP, Brasil

**Keywords:** Prostate, Biopsy, Diagnosis

## Abstract

**Purpose:**

To assess the accuracy of prostate-specific antigen (PSA) adjusted for the transition zone volume (PSATZ) in predicting prostate cancer by comparing the ability of several PSA parameters in predicting prostate cancer in men with intermediate PSA levels of 2.6 – 10.0 ng/mL and its ability to reduce unnecessary biopsies.

**Materials and Methods:**

This study included 656 patients referred for prostate biopsy who had a serum PSA of 2.6 – 10.0 ng/mL. Total prostate and transition zone volumes were measured by transrectal ultrasound using the prolate ellipsoid method. The clinical values of PSA, free-to-total (F/T) ratio, PSA density (PSAD) and PSATZ for the detection of prostate cancer were calculated and statistical comparisons between biopsy-positive (cancer) and biopsy-negative (benign) were conducted.

**Results:**

Cancer was detected in 172 patients (26.2%). Mean PSA, PSATZ, PSAD and F/T ratio were 7.5 ng/mL, 0.68 ng/mL/cc. 0.25 ng/mL/cc and 0.14 in patients with prostate cancer and 6.29 ng/mL, 0.30 ng/mL/cc, 0.16 ng/mL/cc and 0.22 in patients with benign biopsies, respectively. ROC curves analysis demonstrated that PSATZ had a higher area under curve (0,838) than F/T ratio (0.806) (P<0.001) and PSAD (0.806) (P<0.001). With a cut-off value of 0.22 ng/mL/cc, PSATZ had 100% of sensitivity and could have prevented 24% of unnecessary biopsies.

**Conclusions:**

PSATZ may be useful in enhancing the specificity of serum PSA. Compared to other PSA related parameters, it was better in differentiating between prostate cancer and benign prostatic enlargement. Also, PSATZ could reduce a significant number of unnecessary biopsies.

## INTRODUCTION

According to the World Health Organization, prostate cancer (PC) is the second most common cancer and the sixth leading cause of death among males worldwide (1). In 2015, an estimated 27.540 PC-related deaths are anticipated in the United States (1). According to the Brazilian National Cancer Institute (INCA), approximately 68.800 new cases were diagnosed in Brazil in 2014 (2).

Determination of serum levels of prostate-specific antigen (PSA) has been used in clinical practice since 1988, and has become the most valuable tumor marker widely used in screening for prostate cancer. It is considered to be responsible for 45-70% of decreased PC-related deaths reported since 1990 (3). The production of PSA occurs mainly in epithelial cells located in the transition zone (TZ), which makes it organ-specific, but not cancer-specific. Therefore, a multi-parameter approach is essential, given that PSA value taken singularly is not sufficiently accurate, due to the interference of age and frequently coexisting conditions, such as benign prostate hyperplasia (BPH) and prostatitis (4). It seems to be difficult to discriminate between prostate cancer and benign conditions especially among patients with intermediate PSA levels between 2.6 and 10 ng/mL (5). Within this range, there is a trade-off between specificity and sensitivity, a significant degree of the former being lost in the interest of achieving an acceptable degree of the latter (6). In other words, many patients are submitted to unnecessary biopsies because it is important to maintain acceptable diagnosis rates. Therefore, approximately 70% of prostate biopsy results are negative in this group (7). Different PSA parameters such as PSA velocity (6), age specific reference ranges (7), PSA density (PSAD) (8), PSA density adjusted by transition zone (PSATZ) (9) and the correlations between its molecular forms, have been introduced to improve the diagnostic accuracy of serum PSA. However, it remains unclear which method is superior in routine use. Recent studies like PROMIS (10) showed that multiparametric MRI as a first strategy to diagnose PC is effective and cost effective; however, in developing countries such as Brazil, this is not a reality in the great majority of our public health system. Therefore, simpler and most available approaches to evaluate PSA parameters and PC screening are warranted.

The prostate volume (PV) and transition zone volume (TZV) can be determined by transrectal ultrasound (TRUS) using the ellipsoid formula (11). It is known that BPH increases PSA levels by increasing the TZV (4). The ratio between the absolute value of PSA and PV is designated PSA density (PSAD), and the ratio between the absolute value of PSA and TZV is designated PSA density of the transitional zone (PSATZ). The rationale is that by adjusting PSA values for PV or TZV, the influence related to the nonmalignant portion of the gland, which is believed to account for most of the physiological PSA increase, ought to be reduced (8, 9). Some studies have suggested that PSATZ is more specific than PSAD and its use could, therefore, reduce the number of unnecessary biopsies (12, 13).

In this study, we compared PSA and its parameters to evaluate the accuracy of PSATZ in predicting prostate cancer in patients with total PSA levels between 2.6 and 10.0 ng/mL, and whether it could reduce the number of unnecessary biopsies in this group, without missing positive cases.

## MATERIALS AND METHODS

We prospectively included a total of 656 consecutive patients who presented with PSA levels between 2.6 and 10.0 ng/mL and were referred to the University Hospital São Paulo, from January 2014 to December 2016, of the Federal University of São Paulo, Brazil (HSP/UNIFESP) for prostate biopsy. University Hospital São Paulo is a public hospital, responsible for the assistance of nearly six millions people, almost 35% of São Paulo city population. Information regarding clinical and epidemiological characteristics and PSA levels were retrieved after a thorough chart review. Patients with previous history of prostate cancer, hormonal manipulation, documented urinary tract infection, acute or chronic bacterial prostatitis, previous prostate surgery, recent 5-alpha-reductase inhibitors use and any condition that may affect serum PSA level were not included. This study design was approved by the Research Ethics Committee of our Institution, according to the Declaration of Helsinki. All patients included in this study signed an informed consent.

Serum total PSA concentration and free PSA were determined by an enzyme immunoassay (Roche Diagnostics Corporation, Indianapolis, IN, USA).

Transrectal ultrasonography of the prostate was performed by experienced staff using an EnVisor ultrasound system (Philips Healthcare, Eindhoven, The Netherlands) with a 9 MHz endocavity transducer. The prostate was scanned in multiple transverse and sagittal planes. Triaxial distances at the maximal length, width and height of the prostate and the TZ were measured as previously described (4, 13). Both PV and TZV were calculated using the prolate ellipsoid formula (volume=length x width x height x π/6). PSAD and PSATZ were calculated by dividing the PSA value by the PV and TZV, respectively.

Transrectal ultrasound-guided core biopsies of the prostate were performed using an 18-gauge cutting needle in a spring-loaded biopsy gun (Bard Urological, Covington, GA, USA). All biopsies were performed by experienced physicians. Randomly, 20% of our ultrasound measurements results were compared to MRI results, showing a high concordance rate between the two methods. Systematic sextant biopsies with a total of 12 samples were taken from the peripheral zone comprising six peripheral zone biopsies. When ultrasound revealed focal changes, additional samples were taken and sent for analysis. All specimens were adequate for pathologic diagnosis. Prostate intraepithelial neoplasm or atypia were defined as no malignancy.

The results for the quantitative variables are presented as the mean values ± standard error. Variables of different groups were compared using the Mann-Whitney U test. In order to draw comparisons between the patients with cancer and those without, we analyzed the following variables: age; PSA; PV; TZV; PSAD and PSATZ. The Student's t-test for independent samples was used in order to compare the two groups of patients with cancer (positive biopsy) and those without (negative biopsy). The significance of the parameters (PSA, PSAD, PSATZ and F/T ratio) for predicting prostate cancer were assessed based on receiver operating characteristic (ROC) curves, which are plots of the true positive rates (sensitivity) versus the false positive rates (1-specificity), using all different possible cut-off values. The software IBM SPSS - Statistical Package for the Social Sciences, version 22.0 for Windows (SPSS Inc., Chicago, IL, USA) was used in this study.

## RESULTS

Mean patient age was 67.8 (range 46 to 87 years old). Of 656 patients, 172 (26.2%) had a positive biopsy for prostate cancer and 484 (73.8%) had negative biopsies. Table-1 summarizes the features of the distribution of quantitative variables: age, prostate volume; TZ volume; total PSA levels; F/T ratio; PSAD; and PSATZ for patients with positive or negative biopsies. All patients with prostate cancer had significantly higher levels of total PSA, PSAD and PSATZ and significantly lower F/T ratios, PV and TZV when compared to patients with negative biopsies.

**Table 1:** Characteristics of all patients included in the study.

	Total	Cancer	Benign	P-value
N	656	172	484	
Age (years)	67.8±7.09	69.4±6.87	67.6±7.16	0.319
PV (cc)	46.8±22.6	38.5±11.9	48.1±22.5	<0.001
TZV (cc)	24.0±14.3	15.8±7.6	22.7±15.3	<0.001
PSA (ng/mL)	6.61±1.85	7.50±1.70	6.29±1.81	<0.001
F/T ratio	0.20±0.08	0.14±0.05	0.22±0.08	<0.001
PSAD (ng/mL/cc)	0.18±0.06	0.25±0.05	0.16±0.05	<0.001
PSATZ (ng/mL/cc)	0.34±0.15	0. 68±0.12	0.30±0.14	<0.001

**n** = number of patients; **PV** = total prostate volume (cc); **TZV** = transition zone volume (cc); **PSA** = prostate-specific antigen (ng/mL); **F/T** = free-to-total PSA ratio; **PSAD** = PSA density (ng/mL/cc); **PSATZ** = PSA transition zone volume (ng/mL/cc). Data are expressed as mean ± standard deviation

Sensitivity, specificity, positive and negative predictive values and the number of potentially reducible biopsies of each PSA-related parameter were calculated for all 656 patients at different cut-off values (Table-2). A PSAD with a cut-off value of 0.15 ng/mL/cc had a sensitivity of 91% and a specificity of 50%. A PSATZ with a cut-off value of 0.33 ng/mL/cc detected 138 of 172 cancers (80.2%) with a specificity of 69.4%. A F/T ratio with a cut-off value of 0.15 detected 135 of 172 patients (78.5%) with a specificity of 71.1%. In this group, PSATZ provided better results concerning sensitivity, specificity and positive predictive values than PSA, PSAD, PSATZ and F/T ratio. Using a cut-off value for PSATZ of 0.33 ng/mL/cc, we found a sensitivity of 80.2% and a specificity of 69.4%. The maximal cut-off values that preserved 100% of sensitivity, in which no cancer would be missed, were 0.10 ng/mL/cc for PSAD, with a positive predictive value of 0.285; 0.22 ng/mL/cc for PSATZ, with a positive predictive value of 0.348; and 0.31 for F/T ratio with a positive predictive value of 0.285. Of all these parameters, at 100% sensitivity, PSATZ had the highest specificity (33.4%) and the highest positive predictive value (0.348).

**Table 2:** Sensitivity, specificity and reducible biopsies for different PSA, PSAD, F/T ratio and PSATZ values in 656 patients.

	Number of biopsies	Number of cancer	Sensitivity	Specificity	Positive predictive value	Negative predictive value	Reducible biopsies
PSA (ng/mL) greater than							
3.0	656	172	1.000	0.017	0.265	1.000	1.22%
4.0	567	168	0.977	0.176	0.265	0.955	13.5%
5.0	523	154	0.895	0.238	0.294	0.865	20.2%
6.0	406	128	0.744	0.426	0.315	0.824	38.1%
7.0	301	118	0.686	0.622	0.392	0.848	54.1%
8.0	179	76	0.442	0.787	0.425	0.799	72.7%
9.0	74	40	0.233	0.930	0.541	0.773	88.7%
F/T ratio less than							
0.10	82	74	0.430	0.983	0.902	0.829	87.5%
0.12	146	101	0.587	0.907	0.692	0.861	77.7%
0.14	237	121	0.703	0.760	0.511	0.878	63.8%
0.15	275	135	0.785	0.711	0.491	0.903	58.0%
0.18	303	145	0.843	0.674	0.479	0.924	53.8%
0.20	367	149	0.866	0.550	0.406	0.920	44.0%
0.22	384	155	0.901	0.527	0.404	0.938	41.4%
0.25	472	161	0.936	0.357	0.341	0.940	28.0%
0.31	603	172	1.000	0.110	0.285	1.000	8.0%
PSAD (ng/mL per mL) greater than							
0.10	603	172	1.000	0.110	0.285	1.000	8.0%
0.11	570	169	0.983	0.171	0.296	0.965	13.1%
0.13	479	165	0.959	0.351	0.344	0.960	26.9%
0.15	397	158	0.919	0.506	0.398	0.946	39.4%
0.17	336	138	0.802	0.591	0.411	0.894	48.7%
0.18	309	125	0.727	0.620	0.405	0.865	52.9%
0.20	214	124	0.721	0.814	0.579	0.891	67.3%
0.22	161	91	0.529	0.855	0.565	0.836	75.4%
PSATZ (ng/mL per mL) greater than:							
0.15	599	172	1.000	0.118	0.287	1.000	8.7%
0.22	494	172	1.000	0.334	0.348	1.000	24.7%
0.25	452	162	0.942	0.401	0.358	0.951	31.1%
0.30	340	151	0.878	0.610	0.444	0.934	48.1%
0.33	286	138	0.802	0.694	0.483	0.908	56.4%
0.35	262	131	0.762	0.729	0.500	0.896	60.0%
0.37	243	128	0.744	0.762	0.527	0.893	62.9%
0.40	230	128	0.744	0.789	0.557	0.897	64.9%
0.46	195	107	0.622	0.818	0.549	0.859	70.2%
0.55	148	94	0.547	0.888	0.635	0.846	77.4%

**F/T ratio** = free-to-total PSA ratio; **PSA** = prostate-specific antigen; **PSAD** = PSA density; **PSATZ** = PSA density of the transition zone

**Figure 1:**
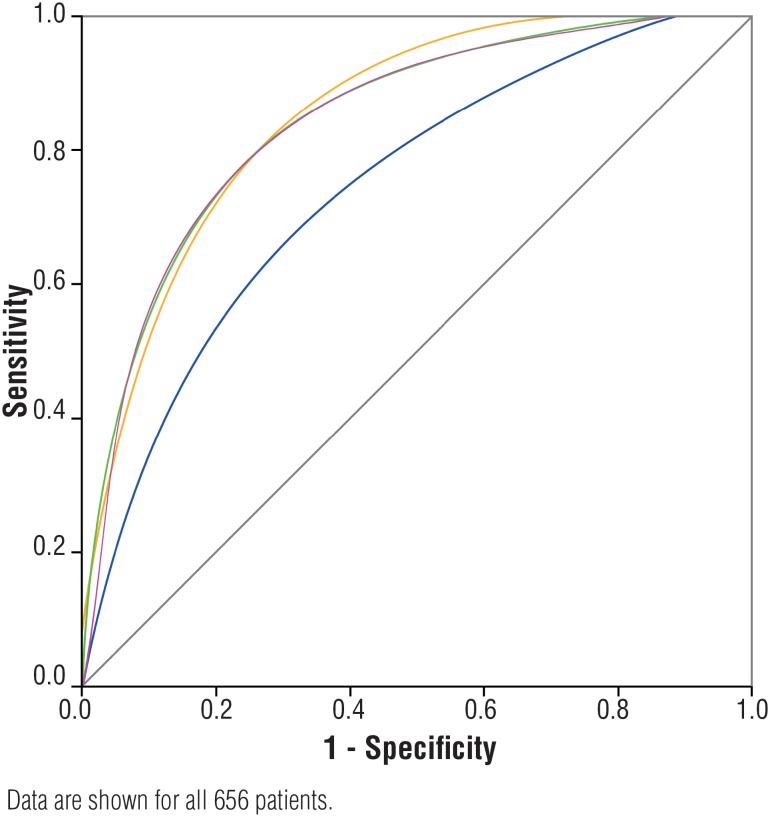
Receiver operating characteristic curves analysis of total prostate specific antigen (PSA)-blue; PSA density for total prostate volume - red; psA density adjusted for transition zone volume - orange; and free-to-total pSA ratio - green; for the detection of prostate carcinoma.

ROC curves analyses were performed in all patients for PSA, PSAD, F/T ratio and PSATZ (Figure-1). At the level of 100% sensitivity, the curve of PSATZ shows better specificity than the others. The AUCs were 0.683 for PSA, 0.806 for PSAD, 0.838 for PSATZ and 0.832 for F/T ratio (Table-3). The AUC of PSATZ was the highest among all of these parameters. Figure-1 demonstrates ROC curves of total PSA, F/T ratio, PSAD and PSATZ.

**Table 3:** Area under Receiver Operating Characteristics Curves of Various PSA Parameters.

Parameter	AUC	P
PSA	0.683	<0.001
PSAD	0.806	<0.001
PSATZ	0.838	<0.001
F/T ratio	0.832	<0.001

**AUC** = area under curve; **PSA** = prostate-specific antigen; **PSAD** = prostate-specific antigen density; **PSATZ** = transition zone prostate-specific antigen density; **F/T ratio** = free-to-total PSA ratio

## DISCUSSION

The role of transrectal prostate biopsy (TRUS-Bx) has changed over time. Its importance has evolved from pure cancer detection to assisting clinical patient management such as active surveillance; however, it is associated with significant morbidity and increased level of anxiety (14). Therefore, maximum efforts should be concentrated to reduce biopsy adverse effects, to improve selection for TRUS-Bx using novel cancer-specific biomarkers and imaging, in an effort to reduce the number of unnecessary biopsies. To address these issues highlighted above, our study underscores the importance of PSATZ as a reliable predictor of prostate cancer for patients with PSA in intermediate levels and its ability to reduce a significant number of unnecessary biopsies. To date, this is the largest Brazilian study addressing these issues.

Determination of serum PSA levels is the most useful available screening test for prostate cancer (6). However, in cases of intermediate PSA levels, it is difficult to discriminate between prostate cancer and BPH, particularly in patients with PSA levels between 2.6 and 10.0 ng/mL, in which there is an overlap between the two conditions (5). To increase the PSA specificity and reduce the number of unnecessary biopsies, which can occur in approximately 70% of the cases, many authors have proposed alternative PSA parameters, such as PSAD (8), PSATZ (9) and the F/T ratio (10). However, there is still some controversy of the use of these PSA parameters in routine use. In this study, we compared some PSA parameters in order to find the most sensitive and specific method to diagnose prostate cancer.

The majority of PSA in serum is bound to protease inhibitors such as ACT (α-1-antichymotripsin) and only a minority exists in the unbound or free form. The proportion of free PSA (fPSA) is lower in men with prostate cancer than in men with BPH (10). Therefore, investigators proposed the concept of free-to-total PSA ratio for the detection of prostate cancer, in order to differentiate prostate cancer from BPH, especially in patients with intermediate PSA levels (10). Early studies showed that a combination of PSA and F/T ratio improved the specificity from 55% to 73% at a sensitivity level of 90% (15). Using a F/T ratio cut-off value of 0.28, Catalona et al. reported a 90% detection of prostate cancers and 12% reduction of biopsies (16). In our study, F/T ratio with a cut-off value of 0.15 we had a sensitivity of 78.5% and a specificity of 71.1% and could reduce unnecessary biopsies by 58%. For a screening test, at 100% sensitivity, F/T ratio with a cut-off value of 0.31 could reduce unnecessary biopsies by 8%. In our study, F/T ratio had better sensitivity and specificity than PSA but was inferior to PSATZ. In ROC analyses it had a higher AUC than PSA and PSAD, but not higher than PSATZ.

It is well recognized that benign prostatic enlargement can result in serum PSA elevation in the absence of prostate carcinoma (4). Benson et al. (8) introduced the concept of PSAD, which is calculated by dividing the total PSA value by the prostate volume. A PSAD with a cut-off 0.15 ng/ mL/cc provided a more reliable indication for ultrasound-guided biopsy of the prostate than PSA alone without significantly compromising cancer detection (8). Although some authors have reported that PSAD is useful in differentiating between prostate cancer and BPH (7, 8, 16), others have questioned its validity (17). Therefore, it is not clear whether PSAD is of real help when deciding if a patient with intermediate levels of PSA must undergo a prostate biopsy. In our study, PSAD with a cut-off value of 0.15 ng/mL/cc had a sensitivity of 78.5% and a specificity of 71.1%, and was inferior to PSATZ. For a screening test, at 100% sensitivity, PSAD with a cut-off value of 0.10 ng/mL/cc could reduce unnecessary biopsies by 8.0%.

It is known that most cases of BPH result from an increase TZV and most PSA leakage from the prostate into the serum comes from the TZ (5, 18). Some studies of correlation between PSA and zonal volume have revealed that the best predictor of serum PSA level is not total prostate volume but TZV, especially TZ epithelial volume (19). Also, ultrasonography has revealed major differences in the proportion of the TZV compared with PV in men with or without BPH per se, implying significant differences between PSAD and PSATZ (11). They also reported a clear correlation between age and TZV. Therefore, adjusting the PSA density for TZV could be a more valuable method than calculating PSAD. Kalish et al.(9) introduced the concept of total PSA adjusted for TZV and suggested that compared with total PSA and PSAD, it was the only significant multivariate predictor using stepwise logistic regression analysis. However, different from our study, they did not adopt ROC analyses as a statistical method, their biopsies were directed at sonographically suspicious areas and they did not include sextant biopsies. Zlotta et al. (20) have also shown that PSATZ was superior to PSAD using ROC analysis, but their study, with fewer patients, was not prospective. They reported the superiority of PSATZ with a cut-off value of 0.35 ng/mL/cc over PSAD and F/T ratio in predicting prostate cancer. In a study involving 281 patients, Kikuchi et al. (21) classified PSATZ as the best method, improving the accuracy of PSA test when compared to total PSA, PSAD and its molecular forms, including fPSA. However, Kobayashi et al. (22) evaluated patients with PSA levels between 2.6 and 4.0 ng/ mL and reported no significant difference between PSAD and PSATZ in terms of their accuracy in detecting prostate cancer. In a recent study, Amini et al. (23) studied the predictability of PSATZ in the diagnosis of prostate cancer among patients with chronic inflammation of prostate and showed a strong correlation between a low PSATZ and the absence of prostate malignancy in this group.

In our study, PSATZ was compared with total PSA, PSAD and F/T ratio in a group of 656 men with PSA levels of 2.6 – 10.0 ng/mL. The AUC of PSATZ was the greatest among all AUCs. The ROC curve of PSATZ deviated to left side, especially at the level of 100% sensitivity, compared with other PSA related parameters. It means that PSATZ could be used as a good screening test. With a cut-off value of 0.22 ng/ mL/cc, we had 100% sensitivity and could have avoided 24.7% of unnecessary biopsies. Therefore, it is reasonable to suggest that PSATZ is, in this study, superior to PSAD and F/T ratio in distinguishing benign from malignant cases, and could be used as an additional PSA parameter to our Brazilian prostate cancer screening program. According to our results, patients with low PSATZ values could possibly be followed less frequently and less aggressively treated.

It is not entirely clear the reasons for the variance in PSATZ reports, but some limitations may include the difficulty of accurate TZV measurement by transrectal ultrasound (TRUS), variability of PSA with aging and variable distribution of glandular and stromal components in BPH (5, 18). The accuracy of TZV measurement is ultra-sonographer dependent, which may influence the reproducibility of PSATZ. It is sometimes difficult to assess the TZV measurement in patients with a very small or very large prostate or with diffuse calcifications, because TZ borders can be less clear in these patients. However, Zlotta et al. (24) showed that, in patients with BPH, when the TZ was measured by an experienced ultrasonographer, there was little difference between the PV and TZV estimated by preoperative transrectal ultrasound and the actual volume of the surgical specimen after prostatectomy. In our study, all TRUS were performed by experienced staff in order to increase inter-operator reliability.

In our data, PSATZ performance results were very similar to F/T ratio. However, the measurement of F/T ratio requires 2 tests and therefore, also increases the sources of bias. Another critical issue relates to the weak stability of fPSA as a protein and results may vary if the samples are not stored at −80°C and/or if they are not analyzed shortly after venipuncture. Djavan et al. detected an intertest variability of >72% when fPSA values from the same patient were drawn in different departments of the same institution (25).

## CONCLUSIONS

In this study, PSATZ was the most reliable test to discriminate between patients with and without prostate cancer compared to other PSA related parameters in patients with intermediate PSA levels. Therefore, PSATZ could be used as a valuable test for biopsy candidates, reducing the number of unnecessary biopsies, therefore improving the cost effectiveness for detecting prostate cancer.
